# Pathogenic assessment of avian influenza viruses in migratory birds

**DOI:** 10.1080/22221751.2021.1899769

**Published:** 2021-03-30

**Authors:** Eun-Ha Kim, Young-ll Kim, Se Mi Kim, Kwang-Min Yu, Mark Anthony B. Casel, Seung-Gyu Jang, Philippe Noriel Q. Pascua, Richard J. Webby, Young Ki Choi

**Affiliations:** a College of Medicine and Medical Research Institute, Chungbuk National University, Cheongju, Korea; b Zoonotic Infectious Diseases Research Center, Chungbuk National University, Cheongju, Korea; c Virology Division, Department. of Infectious Diseases, St. Jude Children’s Research Hospital, Memphis, TN, USA

**Keywords:** Avian influenza virus, wild birds, pathogenicity, transmission, receptor binding

## Abstract

Several subtypes of avian influenza (AI) viruses have caused human infections in recent years; however, there is a severe knowledge gap regarding the capacity of wild bird viruses to infect mammals. To assess the risk of mammalian infection by AI viruses from their natural reservoirs, a panel of isolates from 34 wild birds was examined in animal models. All selected AI virus subtypes were found to predominantly possess Eurasian lineage, although reassortment with North American lineage AI viruses was also noted in some isolates. When used to infect chickens, 20 AI isolates could be recovered from oropharyngeal swabs at 5 days post-infection (dpi) without causing significant morbidity. Similarly, mild to no observable disease was observed in mice infected with these viruses although the majority replicated efficiently in murine lungs. As expected, wild bird AI isolates were found to recognize avian-like receptors, while a few strains also exhibited detectable human-like receptor binding. Selected strains were further tested in ferrets, and 15 out of 20 were found to shed the virus in the upper respiratory tract until 5 dpi. Overall, we demonstrate that a diversity of low-pathogenic AI viruses carried by wild migratory birds have the capacity to infect land-based poultry and mammalian hosts while causing minimal signs of clinical disease. This study reiterates that there is a significant capacity for interspecies transmission of AI viruses harboured by wild aquatic birds. Thus, these viruses pose a significant threat to human health underscoring the need for continued surveillance.

## Introduction

Avian influenza (AI) viruses, classified in the *Orthomyxoviridae* family of RNA viruses, are naturally harboured by wild aquatic birds [1]. AI viruses can be classified into various subtypes by the intrinsic antigenic properties of their surface glycoproteins, hemagglutinin (HA), and neuraminidase (NA) [1]. To date, 16 HA and 9 NA AI virus subtypes have been identified among wild aquatic birds [2] and new strains (H17-18 and N10-11) have been identified in bat species [3].

AI viruses are generally considered to exhibit strong host species preferences and are not readily transmissible to other species. However, the 8-segmented nature of the viral genome and faulty replication mechanisms promote frequent reassortment events and genetic variability, resulting in genetically diverse and novel genomic constellations in wild bird populations. A consequence of this unstable genomic make-up is the potential for transmission and establishment of AI viruses in other animal species. Hence, a wide array of AI strains has been found in sea mammals, terrestrial poultry, horses, dogs, pigs, and importantly, humans [4]. Migratory wild waterfowl generally carry numerous low-pathogenicity AI (LPAI) viruses, which they can transmit along their migratory pathways [1]. Notably, certain LPAI viruses can mutate into lethal highly pathogenic AI (HPAI) virus forms under natural conditions, which commonly occurs upon introduction into high-density host populations (e.g. poultry farms) as in the case of some H5 and H7 virus strains [5].

Prior to 1997, AI virus surveillance and characterization were greatly under-appreciated; however, concern for spillover of AI viruses to human populations has significantly increased since the first reports of human infections with the HPAI A(H5N1) virus in 1997. From 2003 to September 2020, there have been 861 human infections with A(H5N1) recorded by the World Health Organization worldwide, of which approximately 53% were fatal [6]. Moreover, there have been reports of fatal human infections with a novel avian-origin A(H7N9) virus in eastern China since late March 2013 [7]. Recently, this virus was proposed as a major candidate with the potential to emerge as a pandemic in humans [8]. With their proven capacity to infect humans and the likelihood to cause the next pandemic, many studies have been primarily focused on AI viruses within the H5 and H7 subtypes. However, in recent years there has been an increase in reports of avian-to-mammalian infections by various AI subtypes, including H6N1 (A/Taiwan/2/2013) and H10N8 (A/Jiangxi-Donghu/346/201), implying that viruses in addition to the H5 and H7 strains may pose public health risks. With respect to experimental conditions, several studies have also shown that a spectrum of AI virus subtypes can directly infect mammalian hosts [8,9]. Therefore, the characterization of AI viruses isolated from their natural hosts is critical to understand their pathogenic and pandemic potentials.

Given the growing concerns over emerging novel genotypes and the pandemic potential of recent avian influenza viruses, in this study, we characterize the genetic and biological properties of various LPAI viruses. These viruses were isolated from wild migratory birds then used to experimentally infect chickens, mice, and ferrets to assess the capability of these viruses to replicate in various hosts and to determine their pathogenic potential.

## Materials and methods

### Sample collection and virus isolation

Fecal samples were obtained from wild birds in major migratory sites of South Korea. The collected samples were suspended in antibiotic solution and thoroughly mixed followed by centrifugation. Subsequently, the supernatants were inoculated into 10-day-old embryonated chicken eggs and incubated for 48 h at 37°C. Virus isolation was confirmed by hemagglutination assay (HA) and multiplex RT-PCR as described previously [10,11]. After one egg passage, all viruses underwent plaque purification as described [12]. Briefly, Madin Darby Canine Kidney (MDCK) cells were seeded on six-well plates and grown until confluent. Ten-fold serial dilutions of the tested samples were performed in duplicate. After a 1 h incubation at 37°C, the inoculum was then removed and a 1:1 mixture of 1.4% agarose and 2X EMEM with TPCK-trypsin at a final concentration of 1 μg/mL was immediately added as an overlay medium. Approximately 5 plaques were picked after 72 h of incubation and were then individually inoculated into SPF eggs. Virus stocks were aliquoted in 1 ml cryogenic tubes and snap frozen at −80°C until use. The 50% egg infectious dose (EID_50_) was determined using the Reed and Muench method [13].

### Genetic and phylogenetic analyses

After the rapid thaw of frozen viruses, viral RNA was extracted using the RNeasy mini kit (Qiagen, Valencia, CA, USA) or TRIzol reagent (Invitrogen, Carlsbad, CA, USA) with the recommended protocol. The extracted RNA was reverse transcribed at 42°C for 60 min using SuperScript II reverse transcriptase (Invitrogen, Carlsbad, CA, USA). PCR reactions were carried out with influenza-specific primers under standard conditions. PCR products were purified by using the Qiaquick PCR purification kit (Qiagen, Valencia, CA, USA) and conducted sequencing analysis (Accession no. MW557536-MW547768, EU819130, txid947939, txid1082759, txid1855242, and txid1855248). DNA and deduced protein sequences were analyzed and compiled with DNAStar 5.0 (DNAStar, Madison, WI, USA). Genetically closely related viruses were identified using the publicly available basic local alignment search tool analysis and multiple sequence alignments were obtained using Clustal_V [14]. Phylogenetic analyses were performed using the neighbor-joining method with the MEGA software (version 7.0) [15].

### Determination of virus replication in chickens and mice

Five-week-old female specific-pathogen-free white Leghorn chickens (CAVac Lab. Co., Ltd., Daejeon, Korea) were used in this study. Groups of six chickens were separately inoculated oronasally with 10^6^ 50% egg infectious dose per milliliter (EID_50_/mL) of each virus. To test for virus transmission, three contact birds were co-housed with the infected hosts starting at 1 dpi and observed daily for 14 days. Infected chickens shared food and water with the direct-contact (DC) birds. Oropharyngeal and cloacal swabs were collected from the inoculated animals on alternate days and from contact birds every day.

BALB/c (H-2d) mice (6-week-old females weighing ≥ 18 g/mouse; Samtaco, Seoul, Republic of Korea) were anesthetized with an intraperitoneal injection of a Zoletil/xylazine mixture (Zoletil 50®, 80 mg/kg, Virbac, France; Rompun®, 20 mg/kg, Bayer HealthCare, Germany). Groups of twenty-two mice were intranasally inoculated with 10^5.5^ EID_50_/50 µL of virus, and lungs were collected from three mice per group at 3, 5, 7, and 9 dpi to examine virus replication kinetics in this host. Lungs were collected and homogenized (1 g/mL) in cold phosphate-buffered saline (PBS) containing antibiotics (0.1% penicillin/streptomycin; Gibco). The supernatants were serially diluted 10-fold and inoculated into eggs for virus titration (log_10_ EID_50_/g). After 48 h, hemagglutination assays were performed using 0.5% turkey red blood cells. To determine the 50% mouse lethal dose (MLD_50_) of the viruses, we inoculated groups of ten mice i.n. with 10-fold serial dilutions containing 10^1^ to 10^6^ EID_50_ of virus in a 30 µL volume. The MLD_50_ was expressed in terms of log_10_ EID_50_. All EID_50_ and MLD_50_ calculations were performed according to Reed and Muench [13]. The remaining 10 inoculated mice were monitored daily for changes in body weight and survival for 14 days.

### Receptor binding assays

The receptor-binding preference of the AI virus isolates was determined using a solid-phase direct virus-binding assay as previously described [16]. Briefly, wild bird AI viruses were bound to fetuin-coated 96-well microplates at 4°C overnight. Polyacrylamide (PAA)-biotin-conjugated glycans Neu5Acα2–3Galβ1–4Glc β1 (α2,3’-SL-PAA-biotin) or Neu5Acα2-6Galβ1-4GlcNAc (α2,6’SLN-PAA-biotin) (Glycotech Corporation, Gaithersburg, MD, USA) were added to influenza-coated plates at varying dilutions and incubated for an additional 4 h. Glycan binding was detected by adding horseradish peroxidase (HRP)-conjugated streptavidin (Invitrogen, Carlsbad, CA, USA), and absorbance at 450 nm was measured via a VICTOR3 1420 multilabel-counter plate reader (Perkin-Elmer, MA, USA). The receptor-binding specificity of the 2009 pandemic H1N1 virus was also determined and compared as a positive control for binding preference to mammalian virus receptors.

### Experimental infection of ferrets

Outbred female ferrets (*Mustela putorius furo*), 16- to 18-weeks-old and weighing 0.5–0.8 kg (ID Bio Co., Cheongju, Korea), were tested for the absence of antibodies to currently circulating influenza viruses (H5, H7, H9, pH1N1, human seasonal H1N1 and H3N2, and all of the viruses used in this study). For pathogenesis and transmission experiments, 10^6.0^ EID_50_/mL of selected AI viruses (H1N1, H3N2, and H3N4) in 1 mL of sterile PBS was instilled intranasally (i.n.) (500 μL/each nostril) into three groups of ferrets (*n* = 3/group) under anesthesia (Zoletil 50®, 80 mg/kg, Virbac, France; Rompun®, 20 mg/kg, Bayer HealthCare, Germany). All remaining contact ferrets were euthanized at 22 dpi (21 days post-contact, dpc) and their blood samples were tested for specific antibodies to a homologous virus with the HI assay as described elsewhere [11]. At 1 dpi, the experimentally inoculated animals were individually cohoused with DC ferrets (*n* = 3). Baseline body weights and temperatures of the animals were recorded prior to infection and monitored daily for 14 days. Nasal washes were collected from the infected ferrets every other day for 9 days beginning at 1 dpi and daily from 1-day post-exposure (1 dpc) in the contact ferrets.

### Ethics statement

All animal experimental protocols performed in this study strictly followed general animal care guidelines mandated under the Guidelines for Animal Use and Care of the Korea Center for Disease Control (KCDC). They were approved by the Laboratory Animal Research Center (approval No CBNUR-1041-16), which is a member of the Institutional Animal Care and Use Committee of Chungbuk National University.

## Results

### Virus isolation and epidemiology

Fecal samples were collected from free-flying residents and wintering migratory wild aquatic waterfowl in South Korea and viruses were isolated by inoculation into specific-pathogen–free 10-day-old embryonated chicken eggs. A total of 16,317 fecal samples were collected from 2005 to 2012 wherein the 445 samples (2.72%) were detected to be positive for avian influenza virus isolation by RT-PCR and partial sequencing as described [10,11]. Plaque purification of all 445 AIV-positive specimens was attempted and resulted in the deposition of 210 purified viruses into our AIV repository stocks. Identical viruses in the same year were ruled out by HA and NA sequencing (Supplementary Table 4). Virus strains (*n* = 102) sharing more than 99% sequence homology with the one selected were eliminated from this study to rule out possible duplication. A total of 133 AIV-positive specimens (29.8%) were suspected as mixed specimens as they showed at least two different sequences in the same segment by sequencing. To rule out any possible reassortment during cell culture all specimens suspected to be a mixture were not included in our repository. To understand the replicative and pathogenic properties of the viruses, we selected 34 LPAI isolates based on the prevalence and in order to cover all possible subtypes from our repository of AI viruses (Table 1). In this study, we detected H1 through H12 and N1 through N9 subtypes, although some HA and NA combinations were not detected in positives isolates, as shown in Supplementary Table 4. Further, AI viruses of the H13, H14, H15, and H16 subtypes were not isolated over the course of this study. In addition, although HPAI A (H5N1) viruses were isolated they were not included in the present study to focus on LPAI virus strains. We showed the prevalence of each strain or subtype in wild birds (Supplemental Table 4). To understand the replicative and pathogenic properties of the viruses, we selected 34 LPAI isolates based on the subtypes and prevalence which covered all possible subtypes from our repository of AI viruses (Table 1) and evaluated their pathogenic potential in chicken and mouse models.

**Table 1.** Surface glycoprotein (HA and NA) homologies.

Virus	Subtype	Collection site	Hemagglutinin homology		Neuraminidase homology	
			Reference virus	%	Reference virus	%
A/Ab/KoreaW336/2008	H1N1	Mangyeong river	A/EgyptianGs/SouthAfrica/AI1448/2007(H1N8)	97.2	A/Dk/Eastern China/1/2008(H6N1)	98.8
A/Ab/KoreaW228/2007	H1N2	Cheonsoman	A/WDk/Korea/SH29/2006(H1N3)	97.7	A/Ab/Korea/w216/2007(H5N2)	98.9
A/Ab/Korea/W107/2006	H1N3	Pungse	A/Dk/Tsukuba/718/2005(H1N1)	98.6	A/Dk/Eastern China/412/2003(H10N3)	97.8
A/Ab/Korea/W431/2010	H1N8	Mihocheon	A/Gs/Italy/296426/2003(H1N1)	95.6	A/avian/Japan/8KI0162/2008(H3N8)	99.4
A/Ab/Korea/W385/2009	H2N3	Mangyeong river	A/WDk/SH17-1/2008(H2N3)	98.9	A/WDk/SH17-1/2008(H2N3)	99.6
A/Ab/Korea/W180/2007	H2N4	Mihocheon	A/Ml/Sweden/58112/2006(H2N9)	98.3	A/Dk/Tsukuba/20/2007(H8N4)	99.0
A/Ab/Korea/W118/2006	H2N9	Cheonsoman	A/Ml/Sweden/58112/2006(H2N9)	98.5	A/N-Sh/Hong Kong/MPC657/2006(H10N9)	99.2
A/Ab/Korea/CN-3/2005	H3N1	Geumgang	A/Dk/Tsukuba/21/2005(H3N1)	98.6	A/Dk/Tsukuba/718/2005(H1N1)	99.6
A/Ab/Korea/KN-2/2005	H3N2	Mangyeong river	A/En/Korea/ESD11/2003(H3N2)	98.5	A/Ab/Korea/CN-2/2004(H3N2)	99.8
A/Ab/Korea/W146/2006	H3N4	Geumgang	A/Dk/Vietnam/G119/2006(H3N8)	98.1	A/ml/Sweden/24/2002(H8N4)	97.7
A/Ab/Korea/W338/2008	H3N6	Mangyeong river	A/Dk/Vietnam/G119/2006(H3N8)	99.0	A/WDk/Korea/SH5-60/2008(H4N6)	99.6
A/Ab/Korea/KN-4/2005	H3N8	Mangyeong river	A/En/Korea/KCA16/2004(H3N8)	99.9	A/Wbf/Korea/KCA16/2003(H3N8)	99.2
A/Ab/Korea/W340/2008	H4N1	Mangyeong river	A/Av/Japan/8KI0184/2008(H4N6)	99.7	A/Dk/Eastern China/1/2008(H6N1)	99.1
A/Ab/Korea/W319/2008	H4N2	Pungse	A/Dk/Taiwan/wb1101/2006(H4N6)	99.1	A/Ab/Korea/W163/2007(H5N2)	99.8
A/Ab/Korea/W187/2007	H4N4	Geumgang	A/Dk/Mongolia/583/2002(H4N7)	97.3	A/Dk/Tsukuba/20/2007(H8N4)	99.2
A/Ab/Korea/W418/2012	H4N6	Pungse	A/Dk/Thailand/CU-11840T/2011	99.3	A/Dk/Hunan/S11200/2012(H4N6)	99.4
A/Ab/Korea/W120/2006	H5N2	Mihocheon	A/Dk/Shimane/19/2006(H5N2)	99.6	A/Ga/SanJiang/16/2006(H5N2)	99.5
A/Ab/Korea/W346/2009	H5N7	Mangyeong river	A/Dk/Tsukuba/536/2006(H5N2)	98.0	A/Mp/Korea/YJD174/2007(H7N7)	99.1
A/Ab/Korea/W237/2008	H6N1	Geumgang	A/Dk/Guangxi/1157/2006(H6N2)	98.9	A/EmperorGs/Alaska/44063-145/2006(H2N1)	98.5
A/Ab/Korea/W09/2005	H6N2	Cheonsoman	A/Dk/Guangxi/1455/2004(H6N5)	99.6	A/Dk/Tsukuba/9/2005(H5N2)	99.8
A/Ab/Korea/W69/2005	H6N5	Cheonsoman	A/Dk/Guangxi/1455/2004(H6N5)	99.2	A/Dk/Guangxi/1455/2004	99.2
A/Ab/Korea/W72/2005	H6N8	Pungse	A/Dk/Guangxi/585/2005(H6N5)	99.2	A/Sn/Shimane/42/1999(H7N8)	97.6
A/Ab/Korea/W44/2005	H7N3	Cheonsoman	A/WDk/Jiangxi/10179/2005(H7N3)	99.5	A/RSdk/Mongolia/P52/2005(H12N3)	99.3
A/Ab/Korea/W152/2007	H7N7	Mangyeong river	A/Dk/Shiga/B149/2007(H7N7)	99.2	A/Dk/Shimane/18/2006(H7N7)	99.0
A/EM/Korea/W410/2011	H7N9	Mangyeong river	A/Dk/Iwate/301012/2012(H7N1)	99.2	A/northern shoveler/Hong Kong/MPL133/ 2010(H2N9)	99.5
A/Ab/Korea/W141/2006	H8N4	Mangyeong river	A/common teal/Netherlands/1/2005(H8N4)	97.1	A/Dk/Hokkaido/HY57/2005(H9N4)	97.2
A/Ab/Korea/W392/2010	H9N1	Pungse	A/Dk/Thailand/CU-8319T/2010(H9N7)	98.7	A/Ml/Hokkaido/24/2009(H5N1)	99.7
A/Ab/Korea/W408/2012	H9N2	Pungse	A/En/Bangladesh/1041/2009(H9N2)	98.0	A/Ga/Altai/1213/2007(H5N2)	98.7
A/Ab/Korea/W124/2006	H10N2	Mihocheon	A/Ml/Sweden/5812/2005(H10N9)	96.9	A/Ga/SanJiang/16/2006(H5N2)	99.3
A/Ab/Korea/W140/2007	H10N4	Mangyeong river	A/Dk/Hokkaido/W87/2007(H10N2)	99.2	A/Dk/Tsukuba/20/2007(H8N4)	98.2
A/Ab/Korea/W145/2006	H10N9	Geumgang	A/Ml/Sweden/5812/2005(H10N9)	97.1	A/N-Sh/Hong Kong/MPC657/2006(H10N9)	99.2
A/Ab/Korea/W157/2007	H11N2	Geumgang	A/N-Pt/Hong Kong/MPC2085/2007(H11N9)	99.6	A/Ab/Korea/W216/2007(H5N2)	98.2
A/Ab/Korea/W160/2007	H11N9	Geumgang	A/N-Pt/Hong Kong/MPC2085/2007(H11N9)	99.6	A/Dk/Hunan/1590/2007(H6N9)	98.6
A/Ab/Korea/W134/2006	H12N5	Mangyeong river	A/Ml/Sweden/343/2002(H12N5)	97.5	A/Dk/Tsukuba/255/2005(H8N5)	99.0

Closely related viruses were identified through BLAST analysis. Percentage sequence homologies were verified by alignment using DNAStar 5.0 software DNAStar 5.0 (DNAStar, Madison, WI, USA). Ab, aquatic bird; Av, ayan; En, environment; Dk, duck; Mdk, mallard duck; WDk, wild duck; Ml, mallard; N-Sh, northern shoveler; Wbf, wild bird fowl; Ga, garganey; Mp, Magpie; Sn, swan; EmperorGs, emperor goose; RSdk, ruddy shelduck; N-Pt, northern pintail; EgyptianGs, Egyptian goose.

### Genetic characterization

Table 1 shows the reference virus strain bearing the highest nucleotide sequence homology within the surface glycoprotein gene segments to that of the AI viruses selected in this study. Sequence homologies of the HA and NA genes suggest that most of the wild bird isolates are closely related to AI viruses of the Eurasian lineage. However, the HA gene of A/Ab/Korea/W237/2008 (H6N1) is closely related (98.9%) to a 2006 A(H6N2) virus from China (A/Duck/Guangxi/1157/2006 (H6N2)) while the NA gene is most closely related (98.5%) with a North American lineage AI virus represented by (A/emperor goose/Alaska/44063-145/2006 (H2N1)). Similar to HA and NA, sequence analyses of internal viral genes demonstrated that all of the selected AI viruses bear segments derived from AI strains of Eurasian lineage, with exception of the PB1 and PA genes (Supplementary Figure 2). Phylogenetic analysis of PB1 genes revealed that A/Ab/KoreaW228/2007 (H1N2) and A/Ab/Korea/W237/2008 (H6N1) are clustered together with North American lineage AI viruses (Supplementary Figure 2B). On the other hand, the PA gene of A/Ab/Korea/W134/2006(H12N5) diverged from the Eurasian lineage to join A/pintail/Alaska/779/2005(H3N8) (Supplementary Table 1, Supplementary Figure 2C). None of the 34 viruses selected appeared to have contributed to the generation of human-infecting A(H6N1), A(H7N9), A(H10N8) or A(H5N6) avian viruses in China [7,17] or the A(H5N8) that first caused poultry outbreaks in South Korea and Japan in 2014.

### Analysis of molecular pathogenicity markers

Consistent with their low-pathogenicity phenotypes in terrestrial poultry, none of the selected viruses contain a putative cleavage site of HPAI (a motif with multiple basic residues) within their mature HA protein (Supplementary Table 2). The presence of putative receptor binding sites in HA suggests that the affinity of these viruses for avian α 2,3-sialic acid receptors is maintained. Further, none of the AI viruses contained previously characterized pathogenic markers in their deduced PB2 proteins [18]. However, 13 isolates were found to possess a serine residue in their PB1-F2 proteins, which contributes to virulence by promoting apoptosis and facilitating secondary bacterial infections (Supplementary Table 3) [19]. An isoleucine residue at position 97 of the acidic polymerase (PA) protein was noted in 3 AI isolates (H2N3 (W385), H4N2(W319), and H9N2 (W408)), and 28 isolates contained serine residue at position 42 of their non-structural protein 1(NS1) (Supplemental Table 3). The PA_97I_ and NS1_42S_ residues have been independently shown to alter the pathogenicity of avian-origin viruses in mouse models [20]. Nevertheless, sequence information revealed that all viral strains maintained susceptibility to leading antiviral drugs targeting the M2 ion-proton channel and NA proteins, which are prescribed for prophylaxis (Supplementary Tables 2 and 3).

### Growth and transmission of wild bird viruses in chickens

To investigate the potential of the selected isolates to be transmitted and propagated in domestic poultry, groups of six SPF chickens were inoculated through the oronasal route with 10^6^ EID_50_/ml of each virus. At 3, 5, and 7 days post-infection (dpi), tracheal and cloacal swabs were collected to assess virus replication in the animals. Of the AIV isolates examined, 23 out of 34 (67.6%) were shed more efficiently in tracheal swabs of experimentally inoculated chickens than in cloacal swabs (Table 2). Strains that established replication in chickens were mostly of the H1 (3 of 4 NA subtypes), H2 (1 of 3 NA subtypes), H3 (5 of 5 NA subtypes), H4 (4 of 4 Na subtypes), H6 (1 of 4 NA subtypes), H7 (3 of 3 NA subtypes), H9 (2 of 2 NA subtypes) and H10 (1 of 3 NA subtypes) subtypes, many of which persisted up to 5 dpi. Notably, Ab/Kor/W152/07 (H7N7) and Ab/Kor/W392/10(H9N1) were shed at substantially higher titers relative to the other isolates in both the tracheal and cloacal swabs until 5 dpi. None of the directly inoculated chickens exhibited severe clinical signs of influenza disease or mortality typically seen with HPAIV infections. In order to assess the transmission of AI viruses in chickens, which mimics what can occur on poultry farms, direct contact experiments were conducted. Three naive chickens were co-housed with a directly infected chicken and were monitored daily for virus shed through swab collections starting at day 1 post-contact (dpc). Ab/Kor/W336/08 (H1N1), Ab/Kor/W228/07 (H1N2), Ab/Kor/W431/10 (H1N8), Ab/Kor/W385/09 (H2N3), and AB/Kor/W152/07 (H7N7) were recovered in all three co-housed chickens as early as 3 dpc whereas the Ab/Kor/CN-3/05 (H3N1), Ab/Kor/KN-2/05 (H3N2), and Ab/Kor/KN-4/05 (H3N8) contact animals were positive for viral shedding at 5 dpc. On the other hand, only one of the three naive chickens co-housed with Ab/Kor/W09/05 (H6N2)-inoculated chickens shed the virus at 5 dpc. Thus, while a majority of the selected AI isolates from our repository of wild aquatic bird viruses have limited capacity to infect and be transmitted by chickens, several variants were identified that possessed the ability to spread within terrestrial poultry.

**Table 2.** Virus replication in chickens.

Virus	Subtype	Mean oropharyngeal/cloacal swab titers (SD)^a^			Contact transmission^b^	
		3 dpi	5 dpi	7 dpi	Day^b^	No./total
Ab/Kor/W336/08	H1N1	2.5 (0.3)/–	2.1 (0.1)/–	–	3 DPC	3/3
Ab/Kor/W228/07	H1N2	2.1 (0.2)/–	3.0 (0.1)/–	–	3 DPC	3/3
Ab/Kor/W107/06	H1N3	–	–	–	–	0/3
Ab/Kor/W431/10	H1N8	1.5 (0.5)/–	2.5 (0.1)/–	–	3 DPC	3/3
Ab/Kor/W385/09	H2N3	2.5 (0.7)/–	3.0 (0.3)/–	–	3 DPC	3/3
Ab/Kor/W180/07	H2N4	1.7 (0.1)/–	–	–	–	0/3
Ab/Kor/W118/06	H2N9	–	–	–	–	0/3
Ab/Kor/CN-3/05	H3N1	2.3 (0.3)/–	1.7 (0.7)/–	–	5 DPC	3/3
Ab/Kor/KN-2/05	H3N2	2.5 (0.3)/–	1.7 (0.5)/–	–	5 DPC	3/3
Ab/Kor/W146/06	H3N4	2 (0.5)/–	1.3 (0.3)/–	–	–	0/3
Ab/Kor/W338/08	H3N6	2.5 (0.1)/–	1.3 (0.3)/–	–	–	0/3
Ab/Kor/KN-4/05	H3N8	2.3 (0.3)/–	1.7 (0.5)/–	–	5 DPC	3/3
Ab/Kor/W340/08	H4N1	1.1 (0.6)/–	2.0 (0.1)/–	–	–	0/3
Ab/Kor/W319/08	H4N2	3 (0.3)/ 1.3(0.5)	1.5(0.3)/1.5(0.5)	–	–	0/3
Ab/Kor/W187/07	H4N4	2.7 (0.3)/–	1.7 (0.5)/–	–	–	0/3
Ab/Kor/W418/12	H4N6	3 (0.2)/1 (0.6)	1.5(0.5)/1.5(0.7)	–	–	0/3
Ab/Kor/W120/06	H5N2	1.7 (0.5)/–	–	–	–	0/3
Ab/Kor/W346/09	H5N7	–	–	–	–	0/3
Ab/Kor/W237/08	H6N1	–	–	–	–	0/3
Ab/Kor/W09/05	H6N2	1.5 (0.1)/–	2.0 (0.1)/–	–	5 DPC	1/3
Ab/Kor/W69/05	H6N5	–	–	–	–	0/3
Ab/Kor/W72/05	H6N8	–	–	–	–	0/3
Ab/KorW44/05	H7N3	2.3 (0.3)/–	1.7 (0.7)/–	–	–	0/3
Ab/Kor/W152/06	H7N7	2.0(0.1)/3.0(0.5)	2 (0.3)/2.0 (0.7)	–	3 DPC	3/3
A/EM/Kor/W410/11	H7N9	1.7 (0.5)/–	2 (0.3/–)	–	–	0/3
Ab/Kor/W141/06	H8N4	–	–	–	–	0/3
Ab/Kor/W392/10	H9N1	3(0.3)/ 2.5(0.7)	2.5 (0.3)/ 3 (0.5)	–	–	0/3
Ab/Kor/W408/12	H9N2	1 (0.5)/1.3 (0.3)	1.5(0.3)/0.7(0.7)	–	–	0/3
Ab/Kor/W124/06	H10N2	–	–	–	–	0/3
Ab/Kor/W140/07	H10N4	2.3 (0.3)/–	1.3 (0.7)/–	–	–	0/3
Ab/Kor/W145/06	H10N9	–	–	–	–	0/3
Ab/Kor/W157/07	H11N2	–	–	–	–	0/3
Ab/Kor/W160/07	H11N9	–	–	–	–	0/3
Ab/Kor/W134/06	H12N5	1.3 (0.5)/–	–	–	–	0/3

^a^ Titers are mean from six chickens and expressed as log_10_ EID_50_/ml; dashed lines indicate negative virus detection (limit of <0.75 log_10_ EID_50_/ml).

^b^ Earliest timepoint at transmission.

Transmission to cohoused chickens was also determined by monitoring viral titration of oropharyngeal/cloacal swabs; DPC, days post-contact; –, virus not detected.

### Replication and pathogenesis of wild bird viruses in mice

To determine the ability of the selected wild bird AIV isolates to infect and cause disease in mammalian hosts, we experimentally inoculated 10^5.5^EID_50_/50ul of each virus strain intranasally into groups of Balb/c mice. None of the tested viruses induced significant signs of morbidity or mortality in mice more than 6.0 of the 50% mice lethal dose (MLD_50_) monitored daily for 14 days indicating low pathogenicity in this host. One H1 isolate, Ab/Kor/W336/08 (H1N1), induced an average reduction in body weight of 10.2% relative to initial measurements, although none of the mice succumbed to death during the duration of the observation period. All other AI isolates induced mild (less than 10% body weight loss) to no clinical signs of disease.

Twelve isolates could be detected until 5 dpi while 17 isolates persisted until 7 dpi with mean peak titers ranging from 1.3 log_10_ EID_50_/g to 5.0 log_10_ EID_50_/g (Table 3). Almost no viruses could be recovered at 9 dpi, except for Ab/Kor/W152/07(H7N7), which was able to persist in mouse lungs. The highest viral titers were typically detected at 5 dpi and ranged from 2.7 log_10_ EID_50_/g to 5.5 log_10_ EID_50_/g. In contrast, Ab/Kor/W187/07 (H4N4), Ab/Kor/W145/06 (H10N9), Ab/Kor/W157/07 (H11N2), Ab/Kor/W160/07 (H11N9), and Ab/Kor/W134/06 (H12N5) could not be recovered from lung tissues at any time point tested, indicating inefficient replication. Interestingly, although Ab/KorW107/06 (H1N3), Ab/Kor/W118/06 (H2N9), Ab/Kor/W346/09 (H5N7), Ab/Kor/W237/08 (H6N1), Ab/Kor/W69/05 (H6N5), Ab/Kor/W72/05 (H6N8), Ab/Kor/W141/06 (H8N4), and Ab/Kor/W124/06 (H10N2) did not produce detectable titers beyond the limit of virus detection in chickens (Table 2), these eight isolates exhibited the ability to proliferate in mice with lung titers as high as 2.3 to 5.1 log_10_ EID_50_/g (Table 3) indicating different species-specific susceptibility.

**Table 3.** Virus replication in mouse lungs.

Virus	Subtype	Lung viral titers (SD)^a^				Mean Wt. Loss (%)^b^	MLD_50_
		3 dpi	5 dpi	7 dpi	9 dpi		
Ab/Kor/W336/08	H1N1	5.1 (0.1)	5.5 (0.3)	4.1 (0.1)	–	10.2	>6.0
Ab/Kor/W228/07	H1N2	4.5 (0.1)	5.5 (0.3)	4.3 (0.1)	–	4.4	>6.0
Ab/Kor/W107/06	H1N3	2.3 (0.5)	3.3 (0.1)	–	–	1.5	>6.0
Ab/Kor/W431/10	H1N8	5.3 (0.3)	5.5 (0.1)	3.1 (0.5)	–	0.7	>6.0
Ab/Kor/W385/09	H2N3	3.7 (0.3)	4.8 (0.1)	2.2 (0.7)	–	1.3	>6.0
Ab/Kor/W180/07	H2N4	3.3 (0.3)	4.5 (0.5)	1.7 (0.3)	–	3.6	>6.0
Ab/Kor/W118/06	H2N9	3.0 (0.5)	3.7 (0.3)	–	–	0.9	>6.0
Ab/Kor/CN-3/05	H3N1	3.3 (0.3)	4.3 (0.3)	2 (0.5)	–	1.8	>6.0
Ab/Kor/KN-2/05	H3N2	3.5 (0.5)	4.7 (0.5)	2.3 (0.3)	–	2.4	>6.0
Ab/Kor/W146/06	H3N4	4.0 (0.5)	5.0 (0.7)	2.5 (0.5)	–	4.3	>6.0
Ab/Kor/W338/08	H3N6	3.5 (0.3)	4.5 (0.3)	1.7 (0.3)	–	3.5	>6.0
Ab/Kor/KN-4/05	H3N8	3.3 (0.3)	4.3 (0.3)	–	–	2.1	>6.0
Ab/Kor/W340/08	H4N1	5.1 (0.1)	4.5 (0.1)	–	–	2.3	>6.0
Ab/Kor/W319/08	H4N2	3.7 (0.3)	4.5 (0.3)	1.5 (0.3)	–	–	>6.0
Ab/Kor/W187/07	H4N4	–	–	–	–	–	>6.0
Ab/Kor/W418/12	H4N6	2.3 (0.1)	3.5 (0.1)	–	–	0	>6.0
Ab/Kor/W120/06	H5N2	3.5 (0.1)	4.5 (0.30)	2.3 (0.5)	–	5.3	>6.0
Ab/Kor/W346/09	H5N7	3.3 (0.1)	1.5 (0.5)	–	–	2	>6.0
Ab/Kor/W237/08	H6N1	6.1 (0.3)	5.1 (0.2)	3.7 (0.7)	–	7	>6.0
Ab/Kor/W09/05	H6N2	4.1 (0.3)	3.1 (0.1)	2.8 (0.5)	–	4.8	>6.0
Ab/Kor/W69/05	H6N5	3.3 (0.5)	4.5 (0.7)	1.3 (0.3)	–	3.2	>6.0
Ab/Kor/W72/05	H6N8	3.0 (0.3)	2.7 (0.5)	–	–	1.1	>6.0
Ab/Kor/W44/05	H7N3	3.3 (0.3)	5.3 (0.3)	1.7 (0.3)	–	5.3	>6.0
Ab/Kor/W152/06	H7N7	3.0 (0.3)	4.5 (0.3)	5.0 (0.7)	2.5 (0.5)	8.0	>6.0
A/EM/Kor/W410/11	H7N9	3.3 (0.3)	3.0 (0.1)	–	–	4.3	>6.0
Ab/Kor/W141/06	H8N4	2.7 (0.5)	2.7 (0.5)	–	–	2.1	>6.0
Ab/Kor/W392/10	H9N1	3.0 (0.1)	3.7 (0.3)	–	–	3.3	>6.0
Ab/Kor/W408/12	H9N2	2.5 (0.3)	3.5 (0.30)	–	–	2.8	>6.0
Ab/Kor/W124/06	H10N2	3.3 (0.7)	4.7 (0.5)	1.7 (0.5)	–	4.3	>6.0
Ab/Kor/W140/06	H10N4	2.1 (0.5)	0.7 (0.5)	–	–	1.2	>6.0
Ab/Kor/W145/06	H10N9	–	–	–	–	–	>6.0
Ab/Kor/W157/07	H11N2	–	–	–	–	–	>6.0
Ab/Kor/W160/07	H11N9	–	–	–	–	–	>6.0
Ab/Kor/W134/06	H12N5	–	–	–	–	–	>6.0

^a^ Titers are mean lung titers from three mice per time-point and are expressed as log_10_ EID_50_/g; dashed lines indicate negative detection (limit <0.75 log_10_ EID_50_/g).

^b^ Data obtained from 10 mice monitored for 14 dpi.

### Receptor-binding preference profiles

**Figure 1.**
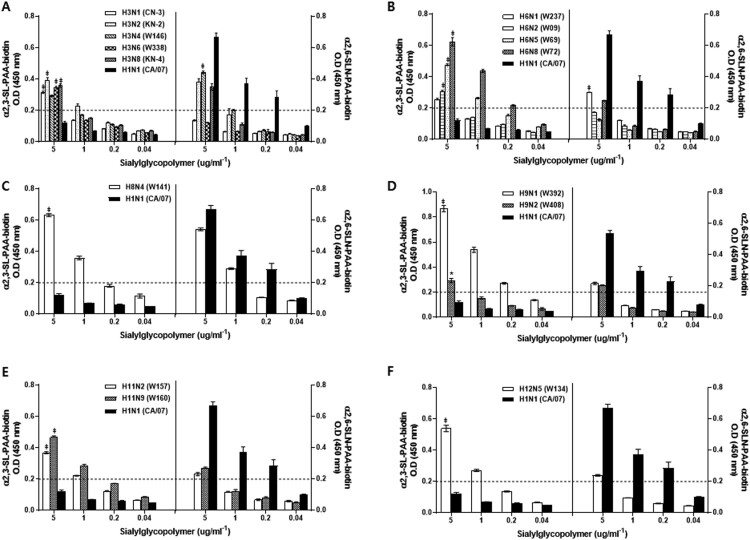
Receptor-binding specificity profiles of H3 (A), H6 (B), H8 (C), H9 (D), H11 (E), and H12 (F) AI virus isolates. Binding affinities of inactivated whole viruses to SA α2,3’-SL-PAA-biotin (left panels) or SA α2,6’SLN-PAA-biotin (right panels) glycans are shown. Results shown are means ± SD (mean of three replicates). Dashed lines indicate the limit of detection. The 2009 pandemic H1N1 virus was used as a positive control for comparing binding preferences for mammalian receptors.

To understand the receptor-binding preferences of each wild bird AIV, we performed solid-phase direct binding assays and measured virus affinity for biotinylated glycans (α 2,3’SA or α 2,6’SLN, which represent the avian and human receptors for these viruses, respectively). We also compared the receptor-binding preference profiles of the isolates with that of A/California/07/2009 [CA/07(H1N1)], the swine-origin pandemic 2009 H1N1 virus, as a reference strain. As expected, the control CA/07(H1N1) virus exhibited poor binding to α2,3-linked sialic acids (α2,3-SAs) but showed a strong binding preference for α2,6-linked sialic acids (α2,6-SAs) even at 0.2 µg/mL^−1^ dilutions of sialyl glycopolymers (Figure 1).

As expected, all 34-wild bird-origin AIVs demonstrated a binding affinity for α 2,3-SAs (Figure 1 and Supplementary Figure 1). However, eleven AIV strains, Ab/Kor/KN-2/05 (H3N2), Ab/Kor/W146/06 (H3N4), Ab/Kor/KN-4/05 (H3N8), Ab/Kor/W237/08 (H6N1), Ab/Kor/W72/05 (H6N8), Ab/Kor/W141/06 (H8N4), Ab/Kor/W392/10 (H9N1), Ab/Kor/W408/12 (H9N2), Ab/Kor/W157/07 (H11N2), Ab/Kor/W160/07 (H11N9) and Ab/Kor/W134/06 (H12N5), also expressed a binding affinity for α2,6-SAs than other viruses (Over limited detection)(Figure 1). Thus, these results suggest that some of the AIVs obtained from wild birds may have the potential to recognize receptors present in mammalian hosts, including humans.

### Pathogenesis and transmission of wild bird viruses in ferrets

To complement the results outlining receptor-binding preferences of these wild bird AI viruses and to investigate their potential threat to humans, we utilized ferrets, which show high susceptibility to human infectious influenza viruses [18]. However, due to the vast list of AIVs characterized here, we narrowed down the virus strains to be screened using the following criteria: (A) viruses that have historically caused human pandemics (i.e. H1N1, H3N2); (B) viruses of purely avian origin previously detected in mammals such as pigs (H2N3, H4N1, H4N6), minks (H10N4), seals (H7N7), and humans (H5N2, H6N1, H7N3, H9N2); and (C) viruses with high binding affinity for α2,6-SAs (over 0.2 O.D) (Figure 1). Groups of ferrets (*n* = 3) were intranasally inoculated with 10^6^ EID_50_/ml of each virus strain and illness progression and virus titers in nasal washes were assessed. Further, to evaluate the potential for ferret-to-ferret transmission by direct contact (DC), naïve contact animals (*n* = 3) were added into the same cage on day 1 post-infection.

Asymptomatic to mildly reduced activity was induced by inoculation with 10^6^ EID_50_/ml of each selected wild bird virus. None of the directly inoculated ferrets exhibited a remarkable reduction generally <15% of body weight loss and <0.7 °C increased in pre-infection baseline temperature [21] in overall body weight or elevation in body temperature (data not shown). Further, there was no evident lethargy or sneezing in these ferrets. In group A, ferrets infected with H1N1and H3N2 AI viruses replicated well and persisted up to 5 dpi (Table 4). In group B, most of the viruses also showed moderate replication and persisted until 5 dpi with the increased virus titers at 3 dpi compared with 1 dpi except H2N3 and H9N2 viruses in all infected ferrets (*n* = 3). Despite frequent reports on human infection cases of land-based avian H9N2 viruses, the Y-439-lineage of H9N2 (W408) showed limited replication property in ferrets. Although the AI viruses of subgroups C showed relatively high affinity for α2,6-SAs (over 0.2 O.D), the infectious H3N4 (W146), H3N8 (KN-4), H6N8 (W72), and H9N1 (W392) were detected until 5 dpi in nasal washes, while the H8N4 and H12N5 viruses were detected in 1 dpi only which may indicate residual virus detection suggesting limited replication in this host.

**Table 4.** Virus replication in the upper respiratory tract of ferrets.

Group	Virus Subtype	Nasal wash titers (log_10_EID_50_/ml)				Contact transmission		Seroconversion
		1 dpi	3 dpi	5 dpi	7 dpi	Day^d^	No./total	No./total
A^a^	Ab/Kor/W336/08 H1N1	3.5 (0.1)	4.4 (0.1)	5.3 (0.1)	–	3DPC	3/3	1/3
	Ab/Kor/KN-2/05 H3N2	3.4 (0.1)	2.5 (0.1)	1.5 (0.5)	–	4DPC	3/3	0/3
B^b^	Ab/Kor/W385/09 H2N3	3.5 (0.1)	2.5 (0.1)	2.0 (0.1)	–	–	0/3	0/3
	Ab/Kor/W340/08 H4N1	2.9 (0.2)	3.4 (0.1)	1.4 (0.2)	–	–	0/3	0/3
	Ab/Kor/W418/12 H4N6	2.5 (0.2)	3.0 (0.2)	1.5 (0.1)	–	–	0/3	0/3
	Ab/Kor/W120/06 H5N2	2.5 (0.2)	3.3 (0.1)	2.5 (0.2)	–	–	0/3	0/3
	Ab/Kor/W237/08 H6N1	2.4 (0.2)	3.3 (0.1)	1.9 (0.2)	–	–	0/3	0/3
	Ab/KorW44/05 H7N3	3.0 (0.2)	3.0 (0.2)	2.0 (0.1)	–	–	0/3	0/3
	Ab/Kor/W152/06 H7N7	2.5 (0.2)	3.5 (0.1)	2.5 (0.1)	–	–	0/3	0/3
	A/EM/Kor/W410/11 H7N9	2.5 (0.2)	3.5 (0.1)	2.0 (0.1)			0/3	0/3
	Ab/Kor/W408/12 H9N2	3.5 (0.1)	1.5 (0.5)	–	–	–	0/3	0/3
	Ab/Kor/W140/07 H10N4	2.5 (0.2)	4.0 (0.1)	2.5 (0.2)	–	–	0/3	0/3
C^c^	Ab/Kor/W146/06 H3N4	3.6 (0.1)	2.9 (0.2)	2.4 (0.2)	–	3DPC	3/3	0/3
	Ab/Kor/KN-4/05 H3N8	2.5 (0.2)	3.3 (0.1)	1.9 (0.2)	–	–	0/3	0/3
	Ab/Kor/W72/05 H6N8	2.0 (0.2)	2.5 (0.2)	1.9 (0.2)	–	–	0/3	0/3
	Ab/Kor/W141/06 H8N4	2.4 (0.2)	–	–	–	–	0/3	0/3
	Ab/Kor/W392/10 H9N1	3.0 (0.2)	3.6 (0.1)	2.5 (0.2)	–	–	0/3	0/3
	Ab/Kor/W157/07 H11N2	2.5 (0.2)	1.5 (0.5)	–	–	–	0/3	0/3
	Ab/Kor/W160/07 H11N9	3.3 (0.1)	1.5 (0.5)	–	–	–	0/3	0/3
	Ab/Kor/W134/06 H12N5	2.5 (0.2)	–	–	–	–	0/3	0/3

^a^ Group of viruses that have historically caused human pandemics (i.e. H1N1 and H3N29).

^b^ Group of viruses of purely avian origin previously detected in mammals such as pigs (H2N3, H4N1, H4N6), minks (H10N4), seals (H7N7), and humans (H5N2, H6N1, H7N3, H7N9, and H9N2).

^c^ Group of viruses with high binding affinity for α2,6-SAs (over 0.2 O.D) (Figure 1).

^d^ Earliest timepoint at transmission.

**Figure 2.**
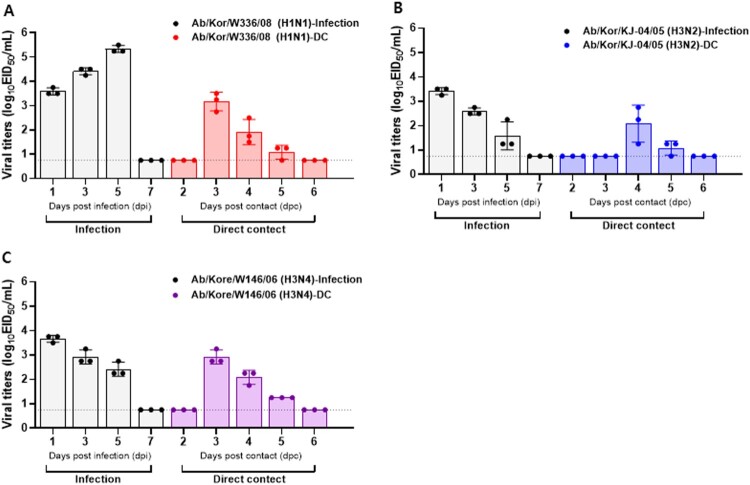
Replication and transmission of AI viruses in ferrets inoculated intranasally with 10^6^ EID_50_/ml of each virus. Individual nasal wash titers of ferrets inoculated with Ab/Kor/W336/08 (H1N1) (A), Ab/Kor/KN-2/05 (H3N2) (B), and Ab/Kor/W146/06 (H3N4) (C). To examine transmission, the inoculated animals were individually paired with direct contact (DC) animals (1:1 setup). Mean viral titers (log_10_ EID_50_/ml) are shown for each group of mammals. The limit of virus detection was 0.75 log_10_ EID_50_/ml.

To assess ferret-to-ferret transmission, nasal washes were collected daily starting on day 1 post-exposure for virus detection and sera were collected at 21 dpc to assess seroconversion. Among the 20 wild bird AI isolates screened, Ab/Kor/W336/08 (H1N1), Ab/Kor/KN-2/05 (H3N2), and Ab/Kor/W146/06 (H3N4) viruses demonstrated the ability to transmit to naïve contact ferrets through direct contact (Figure 2, Table 4). We detected the virus in direct contact ferrets at 3 dpc in H1N1 and H3N4 groups (Figure 2(A,C)), while in the H3N2 group we have confirmed the transmission at 4 dpc (Figure 2(B)). Furthermore, Ab/Korea/W336/08 (H1N1) induced the highest virus shedding in inoculated ferrets with titers peak at 5 dpi as 5.6 log_10_ EID_50_/ml; conversely, the peak viral titers in contact ferrets were significantly lower than those of directly infected ferrets (Figure 2(A)). HI results showed that only one of the H1N1 naïve contact ferrets seroconverted at the end of the experiment although virus was isolated from all of the contact ferrets (Figure 2). These results suggest that an inefficient capacity to replicate in naturally infected ferrets may result in a failure to seroconvert (Figure 2 and Table 4). No other wild bird AI virus was detected in nasal washes of contact ferrets nor did they seroconverted over the course of the experiment.

## Discussion

Due to their ability to cause severe disease with high mortality in poultry and, alarmingly, in humans, HPAI H5 and H7 viruses have been considered to have the greatest potential to cause the next human influenza pandemic. Less lethal LPAI viruses or those that cause asymptomatic infections may have been largely underestimated as they were not previously perceived as threats or precursors for possible pandemics. However, due to heightened awareness over time coupled with improved monitoring techniques and more efficient reporting practices, numerous human infections with various AI viruses have been recorded in recent years, including A(H5N6) [22], A(H6N1) [23], A(H10N7) [24], A(H10N8) [17] and the more widely spread A(H7N9) [25]. Hence, there remains a crucial need to understand the capacity of AI viruses harboured in wild bird reservoirs to infect mammals. In the current study, we genetically and biologically characterized a total of 34 different AI wild bird isolates of varying subtypes collected in known migratory habitats. A barcoding system to identify host species using mitochondrial DNA recovered from fecal samples was not widely implemented in AI virus surveillance prior to 2007 [26,27]. Thus, since in this study AI viruses were isolated using fecal samples collected as far back as 2005, we could not correlate AI prevalence or attribute subtypes to specific bird species. South Korea is a wintering ground, not a breeding site, of adult wild birds such as waterfowl, including mallard ducks [28]. Thus, we can only infer that most of our samples came from mallard ducks.

Phylogenetic analysis revealed that most of our wild bird isolates possessed gene segments commonly found among AI viruses with Eurasian lineage. However, we were also able to identify segments contributed by AIVs of North American lineage suggesting the exchange of gene pools among wild birds. Analysis of AI viruses deposited in public databases showed that reassortment events between these two distinct lineages of AI viruses have been largely rare [29]. Various surveillance studies in South Korea have also reported the sporadic isolation of reassortant wild bird viruses containing segments from North American lineage AI viruses [30]. Increased detection of reassortants may not necessarily be due to increased frequency of reassortment, but rather to more aggressive surveillance for AI activity in the Peninsula. Several studies revealed previously unidentified AI viral genomes completely of North American lineage in Eurasia, or vice versa [31]. However, surveillance along the North Atlantic migratory bird flyways found AIVs of entirely American and Eurasian lineage together with their reassortants in the same geographical region. Since 2014, additional novel H5N8 viruses have caused outbreaks, specifically in the winters of 2016/17 and 2017/18, in South Korea [32]. Migratory birds spread these viruses to Europe and North America as well as to other parts of Asia [33], and the 2014/15 outbreak produced various subtypes (H5Nx viruses) in the United States (21 states) [34]. Therefore, the presence of reassortant viruses indicates the possibility of the worldwide spread of various AI viruses through wild migratory birds.

In addition to pigs, terrestrial poultry, such as chickens, may also act as potential intermediate hosts for the transmission of AI viruses from wild aquatic birds to humans [35]. In groups of experimentally inoculated chickens, about 67.6% of the 34 AI isolates examined were able to establish infection and were efficiently recovered from the upper respiratory tract. Meanwhile, about 26.4% demonstrated direct-contact transmission to co-housed animals suggesting that some of the isolates may have the capacity to be transmitted and become adapted in poultry, or at least in chickens. Accordingly, studies have reported the detection of LPAI H1, H3, H4, H6, and H7 derived from wild birds in domestic poultry [23, 31, 36–38]. It is known that HPAI viruses of the H5 and H7 subtypes originate from their LPAI virus counterparts through the acquisition of polybasic HA cleavage sites upon transmission to domestic poultry [4]. Moreover, it has been shown that several HA subtypes (i.e., H2, H4, and H8) could support the highly pathogenic phenotype when they were modified to contain the virulent HA cleavage site motif [39]. Sequence analysis of viruses recovered from chickens did not reveal the acquisition of the polybasic sequence at the HA cleavage site suggesting that none of the viruses tested in this study could readily evolve to acquire this feature and increase virulence. More importantly, our results show that experimentally inoculated chickens did not manifest significant signs of morbidity commonly caused by HPAI viruses. The asymptomatic nature of infection with these viruses in hosts could become problematic during epidemiologic surveillance efforts. If left undetected, circulation or the repeated introduction of such viruses in domestic poultry would provide ample opportunity for genetic evolution through mutation or reassortment with currently prevailing strains. This could result in the generation of novel AI viruses with an expanded host range similar to what happened with the A(H7N9) virus in China [9].

For practical reasons, such as easier handling, cost-efficiency, and availability of reagents for various assays, the mouse model remains the most widely used to assess the replication and pathogenicity of AI viruses in mammals. In the current study, there was a lack of recognizable clinical disease in infected mice (asymptomatic infection) similar to what was observed in chickens. However, most of the AI isolates in our panel could replicate efficiently in the lungs of mice without prior adaptation. Although similarly mild infections caused by several wild bird isolates from Korea were also observed in BALB/c mice by others [30, 37], a pathogenic H6N5 AI virus was isolated by Nam et al [40] in 2008 was able to spread systemically and caused lethality in mice. For comparison, Driskell et al [9] also reported that a panel of North American wild bird AI virus isolates, which included the HA subtypes H2, H3, H4, H6, H7, and H11, exhibited low-pathogenicity in BALB/c mice despite robust replication in the lungs.

The receptor-binding specificity of HA influences virus replication and transmission. Generally, AI virus subtypes preferentially recognize avian-type α2,3-SA receptors whereas mammalian influenza virus subtypes preferentially bind to α2,6-SA receptors. While all of the wild bird AI viruses in this study demonstrated strong receptor-binding affinities to prototype α2,3-SAs, which was expected due to the lack of essential molecular signatures that alter receptor-binding preferences, 11 strains (32%) of the H3, H6, H8, H9, H11, and H12 HA subtypes also exhibited substantial binding to α2,6-SAs suggesting their potential ability to switch from avian to mammalian hosts. We have observed that the identified H11 (W157 and W160) and H12 (W134) here in the study have no replicative properties in mice. Although we were not able to demonstrate all the AIVs isolated using the ferret model, we found that both H8 (W141) and H12 (W134) were detected at 1 dpi with mean peak titers ranging from 2.4 to 2.5 log10 EID_50_/ml. Moreover, the majority of the AIVs selected exhibited viral replication in the upper respiratory tract which can persist until 5 dpi. Significantly, in this study, we have detected the first H8 wild bird AI virus in Korea [Ab/Kor/W141/06 (H8N4)] and found that despite its relatively high α2,6-SAs binding affinity it lacks efficient replicative properties in ferrets. It would be of best interest to understand Korea isolate H8N4 virus’ host range association and receptor binding affinity.

Further, the Ab/Kor/W336/08 (H1N1), Ab/Kor/KN-2/05 (H3N2), and Ab/Kor/W146/06 (H3N4) isolates demonstrated efficient transmission between ferrets through direct contact suggesting that some H1 and H3 strains circulating in wild birds have the potential to infect and be spread by mammalian hosts. The results of this study demonstrate that H3N2 and H3N4-infected ferrets shed virus up to 5 dpi in the nasal wash, and viral titers were confirmed at 4–5 and 3–5 dpc in each contact group, respectively. However, the serologic test (HI assay) revealed that none of the direct contact ferrets seroconverted. A previous study also showed the absence of seroconversion in ferrets infected with AIV [41]. Further, although the viruses were detected in direct contact ferrets, titers in nasal washes were less than 3.0 log_10_EID_50_/ml and given the short period of viral infection, possibly not high enough to induce the proper antibody response. This could also explain the absence of seroconversion in the contact animals in our study even when the virus was present. It is interesting to note that H3N2 avian-origin A(H3N2) viruses have been reported in dog populations in Korea since 2007 [42] and have also been detected in dogs in Thailand [43]. Although the specific viral progenitors remain unknown, it was proposed that this virus derived from a reassortant AI virus in wild birds. Further, it is noteworthy that both H9 viruses could replicate well in mouse lungs (more than 2.5 log_10_ EID_50_/g (Table 3)), but W392 (H9N1) showed higher virus titers in both chicken and ferrets than did W408 (H9N2) (Tables 2 and 4). Genetic characterizations of these two viruses showed no differences in virulence markers with exception of the PA 97 T/I and NA subtypes. The PA 97I virulence marker was closely associated with mouse adaption of certain avian influenza A viruses [20, 40], and the NA subtype is important for viral fitness due to the balancing of the HA and NA viruses in certain hosts [44]. These data suggest that the high replication properties of the W392 H9N1 virus in chicken and ferrets might be associate with the N1 subtype, while the PA97I substitution in W408 (H9N2) may enable virus replication in mice. Further, both H9 viruses showed moderate replication in infected ferrets; however, transmission to direct-contact animals was not seen in this study. To further understand the detailed mechanisms underlying the differences in growth properties between H9N1 and H9N2 viruses, studies utilizing reverse genetics approaches are needed.

Taken together, a variety of wild bird AI virus subtypes consistently demonstrated their capacity to infect and grow in each of the three animal models tested in this study. In chicken experiments, 9 (26.5%) of 34 AI strains replicated up to 5 dpi and transmitted to direct contact chickens while 29 (85.2%) AI isolates replicated in mouse lungs without the need for prior adaptation. Interestingly, although all tested H1 subtype viruses showed a higher binding affinity for a2,3-SA compared to that for a2,6-SA, all H1 viruses could replicate in mice, but the Ab/Kor/W107/06(H1N3) could not replicate in chickens (Table 2). Of the 11 selected AI isolates (32.4%) with moderate a2,6-SA binding affinities, the H3, H6, H8, H9 subtypes could replicate in the mouse and ferrets without pre-adaption, while the H11 and H12 subtypes could not replicate in mouse lungs (Table 3). Further, when we separated three subgroups based on the criteria in Table 4, only H1N1, H3N2, and H3N4 subtypes of viruses showed relatively high virus titers in all tested animals, demonstrated contact transmissions in ferrets. These results suggest that only limited subtypes of LPAI viruses have the ability to be transmitted from ferret to ferret. Virus replication in certain hosts is affected by many factors including gene mutations and virulence markers as well as receptor binding activity. Thus, predicting the interspecies transmission potential of certain AI viruses is not possible with currently known virological factors, such as receptor binding affinity, and genetic markers. Thus, further study is needed in order to elucidate the interspecies transmission potential of AI viruses.

In summary, we present for the first time a diversity of LPAI viruses carried by wild birds wintering in the South Korean Peninsula, some of which have the capacity to infect land-based poultry and mammalian hosts with minimal signs of clinical disease. To address the need to understand the behaviour of AI viruses in various animal models, our results provide expanded analysis of these viruses making this study the most comprehensive analysis of AI viruses from their principal reservoir. As seen in reports, this study reiterates that AI viruses harboured by wild aquatic birds, subtype notwithstanding, should not be overlooked for their capacity for interspecies transmission. Moreover, these findings should stand to raise awareness and concern about viruses circulating in wild bird populations with the potential to pose serious health risks to both animals and humans. Thus, this underscores the importance of continued virus surveillance in wild aquatic birds.

## Supplementary Material

supplementary_material_editable.docx

Supplementary_Figure_2F.tif

Supplementary_Figure_2E.tif

Supplementary_Figure_2D.tif

Supplementary_Figure_2C.tif

Supplementary_Figure_2B.tif

Supplementary_Figure_2A.tif

Supplementary_Figure_1-revised_version.tif
