# Supplementary material for: Pathogenic assessment of avian influenza viruses in migratory birds
Source: Emerg Microbes Infect. 2021 Mar 30;10(1):565–77. doi: 10.1080/22221751.2021.1899769 (PMC8018353; doi:10.1080/22221751.2021.1899769)
Supplement: supplementary_material_editable.docx [file TEMI_A_1899769_SM0386.docx]

**Supplementary Table 1. Lineage of the internal gene segments of selected wild bird AI viruses.**

| **Virus** | **Subtype** | **Internal Gene segments** | | | | | |
| --- | --- | --- | --- | --- | --- | --- | --- |
|  |  | **PB2** | **PB1** | **PA** | **NP** | **M** | **NS** |
| Ab/Kor/W336/08 | H1N1 | EA | EA | EA | EA | EA | EA |
| Ab/Kor/W228/07 | H1N2 | EA | NAm | EA | EA | EA | EA |
| Ab/KorW107/06 | H1N3 | EA | EA | EA | EA | EA | EA |
| Ab/Kor/W431/10 | H1N8 | EA | EA | EA | EA | EA | EA |
| Ab/Kor/W385/09 | H2N3 | EA | EA | EA | EA | EA | EA |
| Ab/Kor/W180/07 | H2N4 | EA | EA | EA | EA | EA | EA |
| Ab/Kor/W118/06 | H2N9 | EA | EA | EA | EA | EA | EA |
| Ab/Kor/CN-3/05 | H3N1 | EA | EA | EA | EA | EA | EA |
| Ab/Kor/ KN-2/05 | H3N2 | EA | EA | EA | EA | EA | EA |
| Ab/Kor/W146/06 | H3N4 | EA | EA | EA | EA | EA | EA |
| Ab/Kor/W338/08 | H3N6 | EA | EA | EA | EA | EA | EA |
| Ab/Kor/KN-4/05 | H3N8 | EA | EA | EA | EA | EA | EA |
| Ab/Kor/W340/08 | H4N1 | EA | EA | EA | EA | EA | EA |
| Ab/Kor/W319/08 | H4N2 | EA | EA | EA | EA | EA | EA |
| Ab/Kor/W187/07 | H4N4 | EA | EA | EA | EA | EA | EA |
| Ab/Kor/W418/12 | H4N6 | EA | EA | EA | EA | EA | EA |
| Ab/Kor/W120/06 | H5N2 | EA | EA | EA | EA | EA | EA |
| Ab/Kor/W346/09 | H5N7 | EA | EA | EA | EA | EA | EA |
| Ab/Kor/W237/08 | H6N1 | EA | NAm | EA | EA | EA | EA |
| Ab/Kor/W09/05 | H6N2 | EA | EA | EA | EA | EA | EA |
| Ab/Kor/W69/05 | H6N5 | EA | EA | EA | EA | EA | EA |
| Ab/Kor/W72/05 | H6N8 | EA | EA | EA | EA | EA | EA |
| Ab/Kor/W44/05 | H7N3 | EA | EA | EA | EA | EA | EA |
| Ab/Kor/W152/07 | H7N7 | EA | EA | EA | EA | EA | EA |
| A/EM/Kor/W410/11 | H7N9 | EA | EA | EA | EA | EA | EA |
| Ab/Kor/W141/06 | H8N4 | EA | EA | EA | EA | EA | EA |
| Ab/Kor/W392/10 | H9N1 | EA | EA | EA | EA | EA | EA |
| Ab/Kor/W408/12 | H9N2 | EA | EA | EA | EA | EA | EA |
| Ab/Kor/W124/06 | H10N2 | EA | EA | EA | EA | EA | EA |
| Ab/Kor/W140/06 | H10N4 | EA | EA | EA | EA | EA | EA |
| Ab/Kor/W145/06 | H10N9 | EA | EA | EA | EA | EA | EA |
| Ab/Kor/W157/07 | H11N2 | EA | EA | EA | EA | EA | EA |
| Ab/Kor/W160/07 | H11N9 | EA | EA | EA | EA | EA | EA |
| Ab/Kor/W134/06 | H12N5 | EA | EA | NAm | EA | EA | EA |

EA, Eurasian avian-like lineage; NAm, North American avian-like lineage

**Supplementary Table 2. Molecular analysis of surface glycoproteins.**

| **Virus** | **Subtype** | **HA protein (H3 numbering)** | | | | | **NA protein**  **(N2 numbering)** | | |
| --- | --- | --- | --- | --- | --- | --- | --- | --- | --- |
|  |  | **Cleavage site** | **190** | **225** | **226** | **228** | **Stalk Deletion** | **119** | **274** |
| Ab/Kor/W336/08 | H1N1 | PSIQSR/GLF | E | G | Q | G | No | E | H |
| Ab/Kor/W228/07 | H1N2 | PSIQSR/GLF | E | G | Q | G | No | E | H |
| Ab/KorW107/06 | H1N3 | PSIQSR/GLF | E | G | Q | G | No | E | H |
| Ab/Kor/W431/10 | H1N8 | PSIQSR/GLF | E | G | Q | G | No | E | H |
| Ab/Kor/W385/09 | H2N3 | PQIESR/GLF | E | G | Q | G | No | E | H |
| Ab/Kor/W180/07 | H2N4 | PQIESR/GLF | E | G | Q | G | No | E | H |
| Ab/Kor/W118/06 | H2N9 | PQIESR/GLF | E | G | Q | G | No | E | H |
| Ab/Kor/CN-3/05 | H3N1 | PEKQTR/GLF | E | G | Q | G | No | E | H |
| Ab/Kor/ KN-2/05 | H3N2 | PEKQTR/GLF | E | G | Q | G | No | E | H |
| Ab/Kor/W146/06 | H3N4 | PEKQTR/GLF | E | G | Q | G | No | E | H |
| Ab/Kor/W338/08 | H3N6 | PEKQTR/GLF | E | G | Q | G | No | E | H |
| Ab/Kor/KN-4/05 | H3N8 | PEKQTR/GLF | E | G | Q | G | No | E | H |
| Ab/Kor/W340/08 | H4N1 | PEKASR/GLF | E | G | Q | G | No | E | H |
| Ab/Kor/W319/08 | H4N2 | PEKASR/GLF | E | G | Q | G | No | E | H |
| Ab/Kor/W187/07 | H4N4 | PEKASR/GLF | E | G | Q | G | No | E | H |
| Ab/Kor/W418/12 | H4N6 | PEKASR/GLF | E | G | Q | G | No | E | H |
| Ab/Kor/W120/06 | H5N2 | PQRETR/GLF | E | G | Q | G | No | E | H |
| Ab/Kor/W346/09 | H5N7 | PQRETR/GLF | E | G | Q | G | No | E | H |
| Ab/Kor/W237/08 | H6N1 | PQEITR/GLF | E | G | Q | G | No | E | H |
| Ab/Kor/W09/05 | H6N2 | PQEITR/GLF | E | G | Q | G | No | E | H |
| Ab/Kor/W69/05 | H6N5 | PQEITR/GLF | E | G | Q | G | No | E | H |
| Ab/Kor/W72/05 | H6N8 | PQEITR/GLF | E | G | Q | G | No | E | H |
| Ab/Kor/W44/05 | H7N3 | PEIPKGR/GLF | E | G | Q | G | No | E | H |
| Ab/Kor/W152/07 | H7N7 | PEIPKGR/GLF | E | G | Q | G | No | E | H |
| A/EM/Kor/W410/11 | H7N9 | PEIPKGR/GLF | E | G | Q | G | No | E | H |
| Ab/Kor/W141/06 | H8N4 | PSIEPK/GLF | E | G | Q | G | No | E | H |
| Ab/Kor/W392/10 | H9N1 | PAASDR/GLF | E | G | Q | G | No | E | H |
| Ab/Kor/W408/12 | H9N2 | PAASDR/GLF | E | G | Q | G | No | E | H |
| Ab/Kor/W124/06 | H10N2 | PEIMQGR/GLF | E | G | Q | G | No | E | H |
| Ab/Kor/W140/06 | H10N4 | PEIMQGR/GLF | E | G | Q | G | No | E | H |
| Ab/Kor/W145/06 | H10N9 | PEIMQGR/GLF | E | G | Q | G | No | E | H |
| Ab/Kor/W157/07 | H11N2 | PAIASR/GLF | E | G | Q | G | No | E | H |
| Ab/Kor/W160/07 | H11N9 | PAIASR/GLF | E | G | Q | G | No | E | H |
| Ab/Kor/W134/06 | H12N5 | PQAQDR/GLF | E | G | Q | G | No | E | H |

**Supplementary Table 3. Molecular analysis of the internal proteins.**

| **Virus Name** | **Subtype** | **PB2** | | | | | **PB1-F2** | **PA** | **M2** | | **NS1** | | |
| --- | --- | --- | --- | --- | --- | --- | --- | --- | --- | --- | --- | --- | --- |
|  |  | **158** | **271** | **591** | **627** | **701** | **66S** | **97** | **27** | **31** | **Deletion^c^** | **42S** | **C-terminal** |
| Ab/Kor/W336/08 | H1N1 | E | T | Q | E | D | N | T | V | S | NO | S | ESEV |
| Ab/Kor/W228/07 | H1N2 | E | T | Q | E | D | S | T | V | S | NO | S | ESEV |
| Ab/Kor/W107/06 | H1N3 | E | T | Q | E | D | N | T | V | S | NO | S | ESEV |
| Ab/Kor/W431/10 | H1N8 | E | T | Q | E | D | N | T | I | S | NO | S | ESEV |
| Ab/Kor/W385/09 | H2N3 | E | T | Q | E | D | N | I | V | S | NO | S | ESEV |
| Ab/Kor/W180/07 | H2N4 | E | T | Q | E | D | N | T | V | S | NO | A | ESEV |
| Ab/Kor/W118/06 | H2N9 | E | T | Q | E | D | S | T | V | S | NO | S | ESEV |
| Ab/Kor/CN-3/05 | H3N1 | E | T | Q | E | D | S | T | V | S | NO | A | ESEV |
| Ab/Kor/ KN-2/05 | H3N2 | E | T | Q | E | D | S | T | V | S | NO | S | ESEV |
| Ab/Kor/W146/06 | H3N4 | E | T | Q | E | D | N | T | V | S | NO | S | ESEV |
| Ab/Kor/W338/08 | H3N6 | E | T | Q | E | D | N | T | V | S | NO | S | ESEV |
| Ab/Kor/KN-4/05 | H3N8 | E | T | Q | E | D | S | T | V | S | NO | A | ESEV |
| Ab/Kor/W340/08 | H4N1 | E | T | Q | E | D | N | T | V | S | NO | S | ESEV |
| Ab/Kor/W319/08 | H4N2 | E | T | Q | E | D | S | I | V | S | NO | S | ESEV |
| Ab/Kor/W187/07 | H4N4 | E | T | Q | E | D | S | T | V | S | NO | S | ESEV |
| Ab/Kor/W418/12 | H4N6 | E | T | Q | E | D | S | T | V | S | NO | S | ESEV |
| Ab/Kor/W120/06 | H5N2 | E | T | Q | E | D | N | T | V | S | NO | A | ESEV |
| Ab/Kor/W346/09 | H5N7 | E | T | Q | E | D | N | T | V | S | NO | A | ESEV |
| Ab/Kor/W237/08 | H6N1 | E | T | Q | E | D | S | T | V | S | NO | S | ESEV |
| Ab/Kor/W09/05 | H6N2 | E | T | Q | E | D | S | T | V | S | NO | S | ESEV |
| Ab/Kor/W69/05 | H6N5 | E | T | Q | E | D | N | T | V | S | NO | S | ESEV |
| Ab/Kor/W72/05 | H6N8 | E | T | Q | E | D | N | T | V | S | NO | S | ESEV |
| Ab/Kor/W44/05 | H7N3 | E | T | Q | E | D | N | T | V | S | NO | S | ESEV |
| Ab/Kor/W152/07 | H7N7 | E | T | Q | E | D | N | T | V | S | NO | S | ESEV |
| A/EM/Kor/W410/11 | H7N9 | E | T | Q | E | D | N | T | V | S | NO | S | ESEV |
| Ab/Kor/W141/06 | H8N4 | E | T | Q | E | D | N | T | V | S | NO | S | ESEV |
| Ab/Kor/W392/10 | H9N1 | E | T | Q | E | D | N | T | V | S | NO | S | ESEV |
| Ab/Kor/W408/12 | H9N2 | E | T | Q | E | D | N | I | V | S | NO | A | ESEV |
| Ab/Kor/W124/06 | H10N2 | E | T | Q | E | D | N | T | V | S | NO | A | ESEV |
| Ab/Kor/W140/07 | H10N4 | E | T | Q | E | D | N | T | V | S | NO | S | ESEV |
| Ab/Kor/W145/06 | H10N9 | E | T | Q | E | D | N | T | V | S | NO | A | ESEV |
| Ab/Kor/W157/07 | H11N2 | E | T | Q | E | D | S | T | V | S | NO | S | ESEV |
| Ab/Kor/W160/07 | H11N9 | E | T | Q | E | D | N | T | V | S | NO | S | ESEV |
| Ab/Kor/W134/06 | H12N5 | E | T | Q | E | D | S | T | V | S | NO | S | ESEV |
|  |  |  |  |  |  |  |  |  |  |  |  |  |  |

**Supplementary Table 4. Subtypes of avian influenza viruses isolated from 2005 to 2012 in Korea**

^a^ Except subtype of the same year

| **Avian Virus**  **(Percent IAV prevalence)** | **H1** | **H2** | **H3** | **H4** | **H5** | **H6** | **H7** | **H8** | **H9** | **H10** | **H11** | **H12** | **Total** |
| --- | --- | --- | --- | --- | --- | --- | --- | --- | --- | --- | --- | --- | --- |
|  | 18 (8.5%) | 3 (1.42%) | 14 (6.6%) | 17  (8%) | 92 (43.8%) | 12 (5.7%) | 21 (10%) | 4  (1.9%) | 3  (1.42%) | 10 (4.7%) | 12  (5.7%) | 4  (1.9%) | 210^a^ |
| **N1** | 8 |  |  |  | 6 | 2 |  |  |  |  |  |  |  |
| **N2** | 4 |  | 3 | 4 | 34 | 5 |  |  | 3 | 1 | 1 |  |  |
| **N3** | 3 | 3 |  |  | 9 |  | 1 |  |  |  |  | 2 |  |
| **N4** |  |  |  | 1 |  |  |  | 3 |  | 6 |  |  |  |
| **N5** | 1 |  |  |  |  | 2 | 1 |  |  | 1 |  | 2 |  |
| **N6** | 2 |  | 1 | 12 | 10 | 1 |  | 1 |  | 1 |  |  |  |
| **N7** |  |  |  |  | 1 |  | 19 |  |  | 1 |  |  |  |
| **N8** |  |  | 10 |  | 32 | 2 |  |  |  |  |  |  |  |
| **N9** |  |  |  |  |  |  |  |  |  |  | 11 |  |  |
