# Supplementary figures and images for: Pathogenic assessment of avian influenza viruses in migratory birds
Source: Emerg Microbes Infect. 2021 Mar 30;10(1):565–77. doi: 10.1080/22221751.2021.1899769 (PMC8018353; doi:10.1080/22221751.2021.1899769)

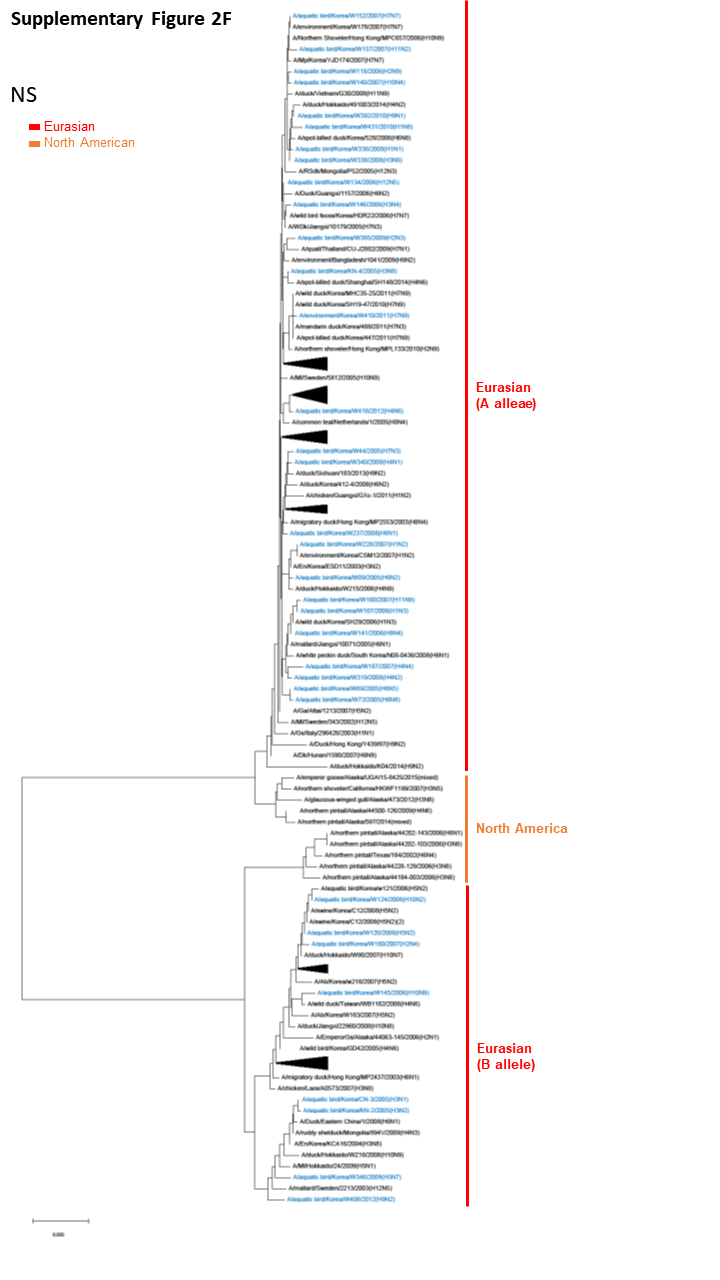

Supplement: Supplementary_Figure_2F.tif [file TEMI_A_1899769_SM0385.tif]

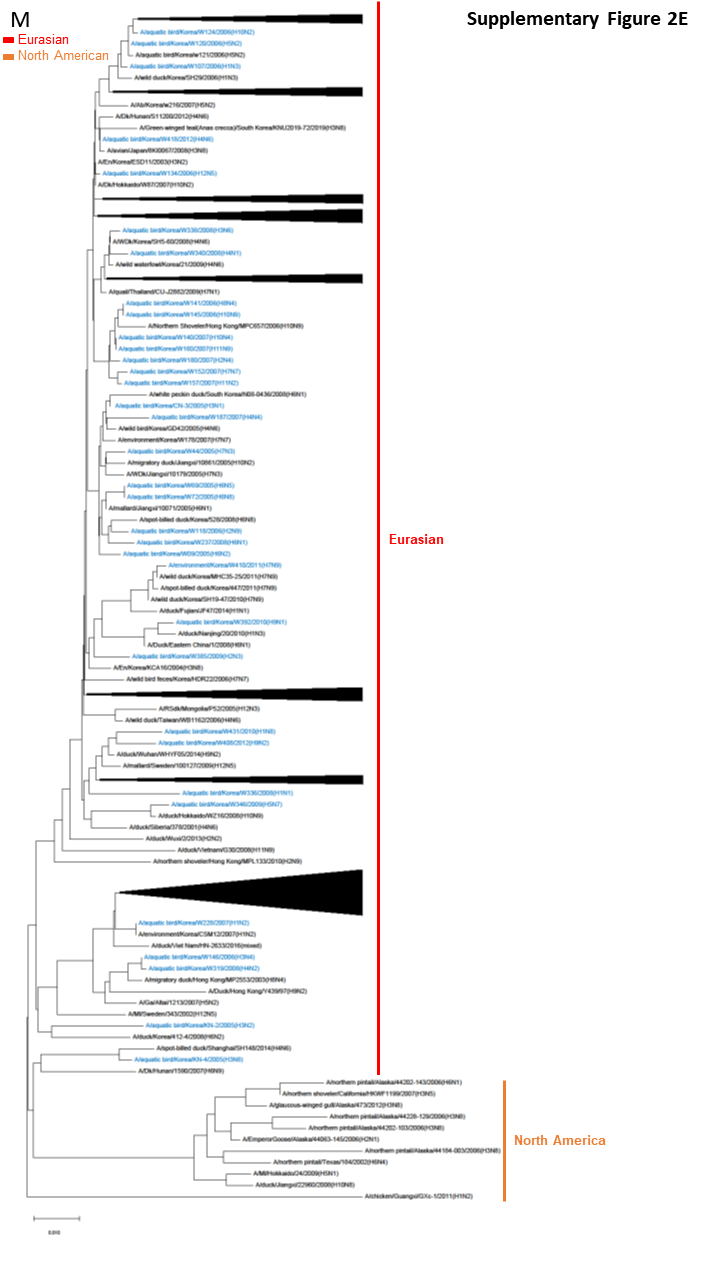

Supplement: Supplementary_Figure_2E.tif [file TEMI_A_1899769_SM0384.tif]

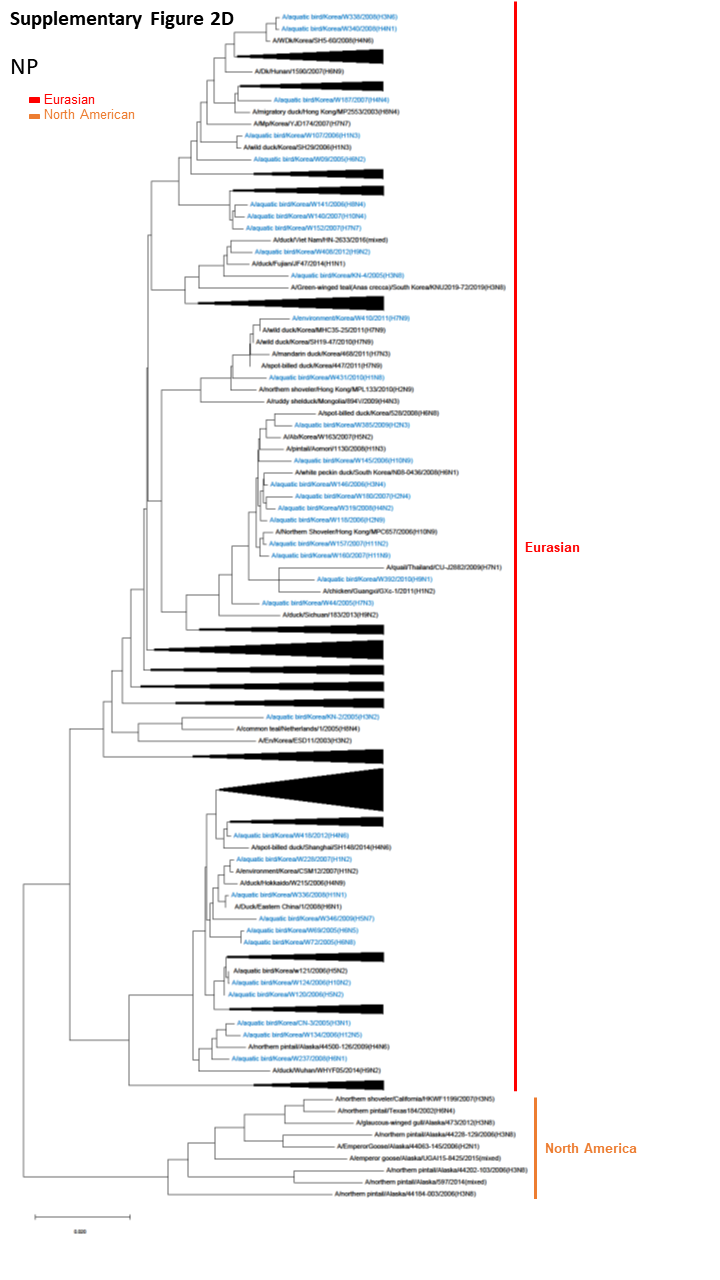

Supplement: Supplementary_Figure_2D.tif [file TEMI_A_1899769_SM0383.tif]

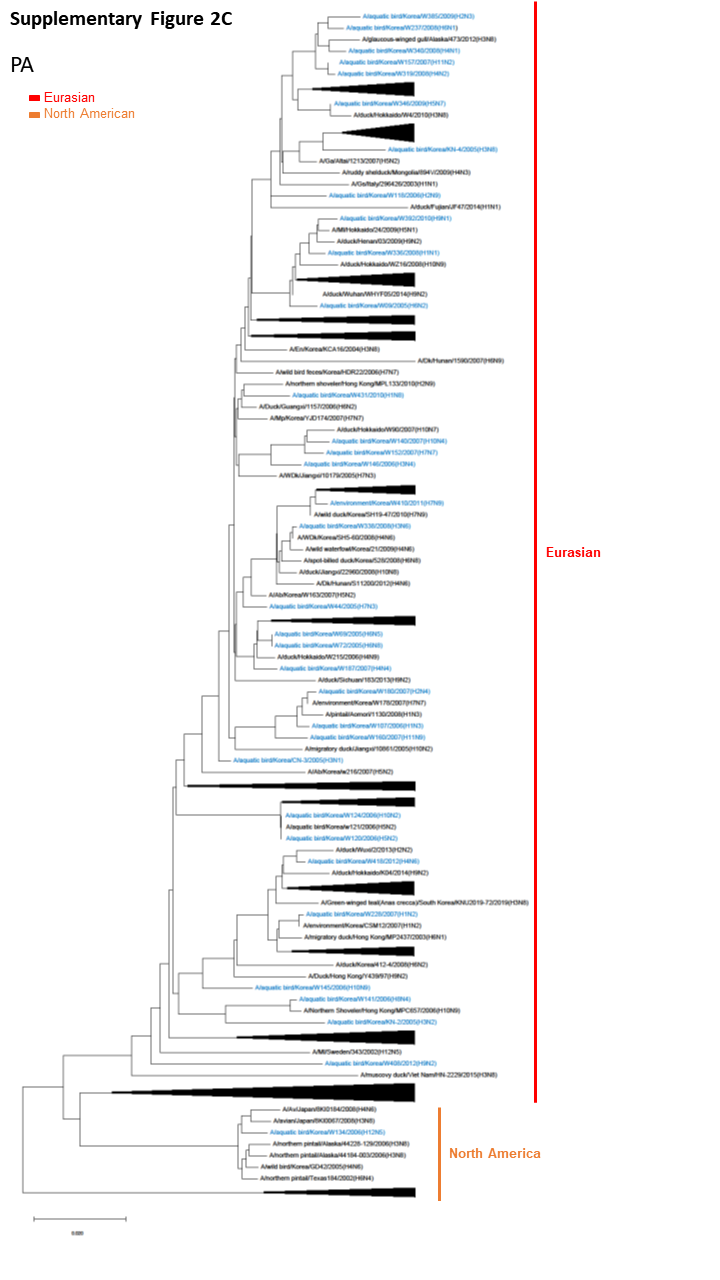

Supplement: Supplementary_Figure_2C.tif [file TEMI_A_1899769_SM0382.tif]

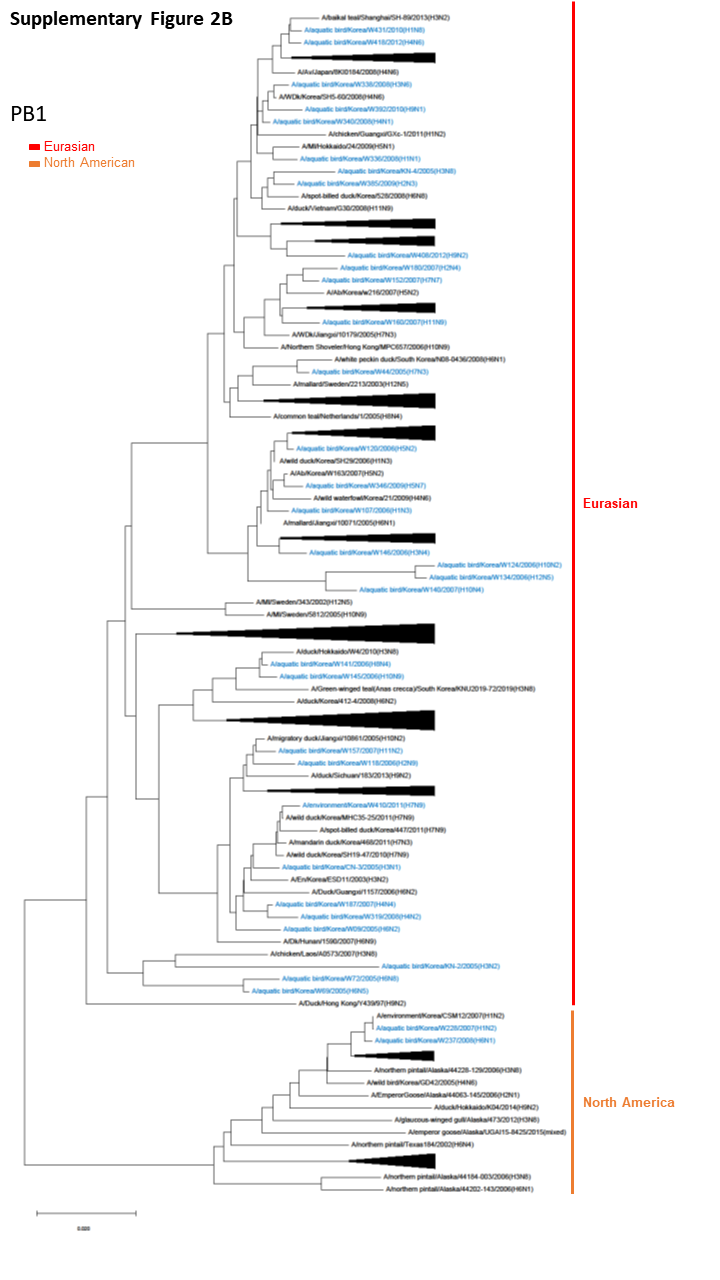

Supplement: Supplementary_Figure_2B.tif [file TEMI_A_1899769_SM0381.tif]

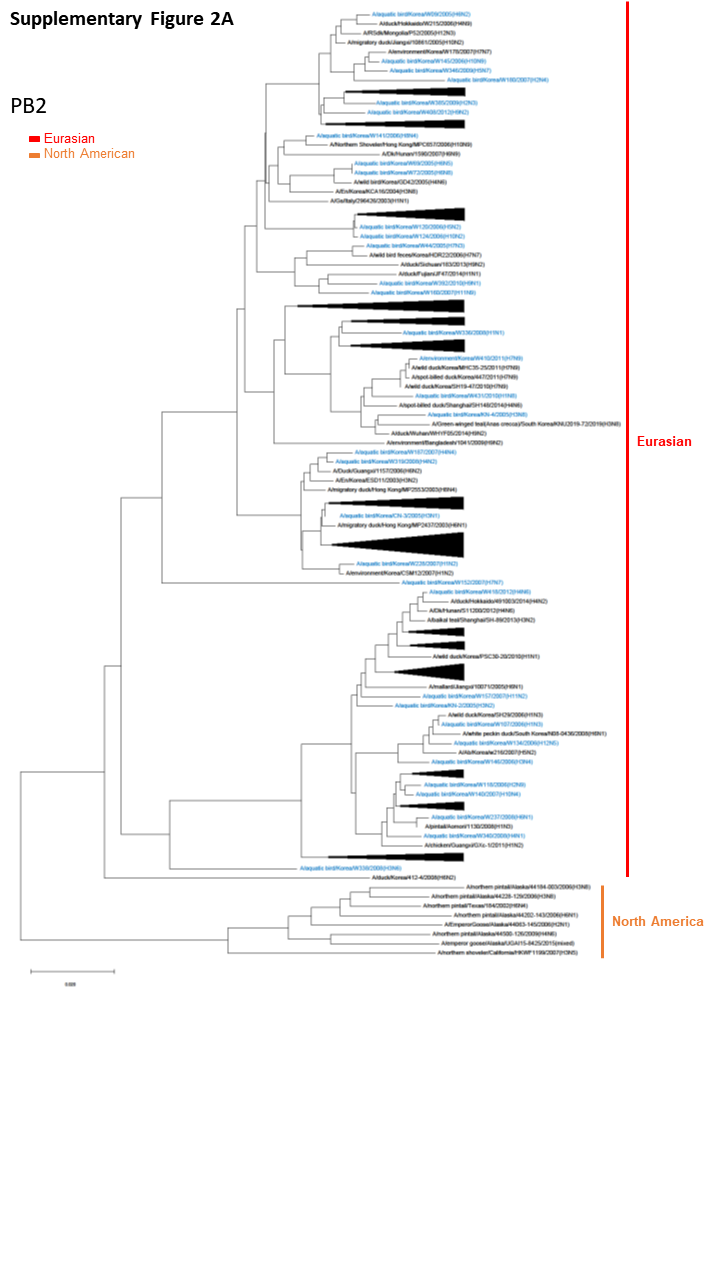

Supplement: Supplementary_Figure_2A.tif [file TEMI_A_1899769_SM0380.tif]

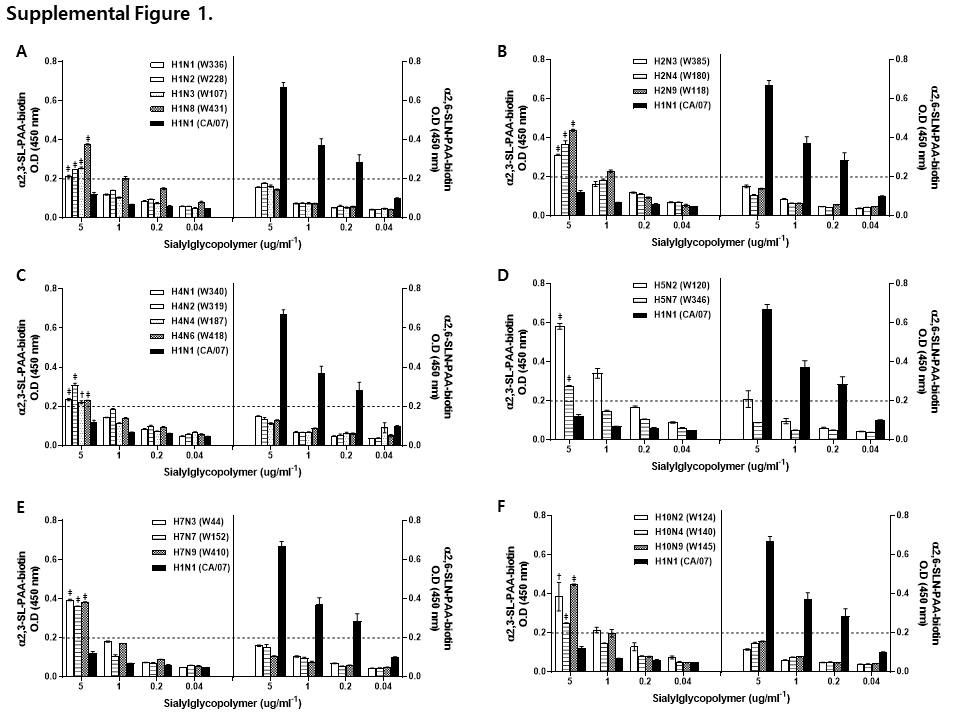

Supplement: Supplementary_Figure_1-revised_version.tif [file TEMI_A_1899769_SM0379.tif]
